# Febrile neutropenia associated with adalimumab in a patient with ulcerative colitis

**DOI:** 10.1002/ccr3.8629

**Published:** 2024-03-27

**Authors:** Ma’In Atallah Mohammad Abumuhfouz, Anas Alsadi, Waseem Fadhl Umer, Mhd Baraa Habib

**Affiliations:** ^1^ Department of Internal Medicine Hamad Medical Corporation Doha Qatar; ^2^ Department of Cardiology Heart Hospital, Hamad Medical Corporation Doha Qatar

**Keywords:** Adalimumab, Crohn's disease, fever, neutropenia

## Abstract

Adalimumab has become essential for managing various chronic inflammatory diseases, including inflammatory bowel disease (IBD). While hematologic complications of adalimumab therapy are rare, they can have significant clinical implications. This report highlights the importance of recognizing and monitoring for neutropenia in patients receiving adalimumab treatment. We also describe the potential mechanisms and management strategies for this adverse event.

## 1 INTRODUCTION

Tumor necrosis factor alpha (TNF‐α), a proinflammatory cytokine, plays a crucial role in the pathogenesis of various chronic inflammatory conditions such as rheumatoid arthritis, spondyloarthropathies, and inflammatory bowel disease (IBD).^1^ The inhibition of TNF‐α signaling through anti‐TNF therapy has emerged as a pivotal and effective treatment approach for patients with severe or refractory IBD.^2^

While hematologic complications associated with anti‐TNF therapy are rare, they do include neutropenia. A post‐marketing study conducted by Feltelius et al.^3^ examined 820 patients receiving etanercept over a 4‐year period and identified hematologic complications in 3.2% of patients, with leukopenia noted in 4 individuals. In another investigation by Miehsler et al., the incidence of leukopenia and neutropenia in patients receiving infliximab was found to be 1.5% and 1.1%, respectively.^4^

Based on the available data, the occurrence of neutropenia in patients undergoing anti‐TNF therapy may have been underestimated. Bessissow et al. conducted a study involving 111 individuals experiencing anti‐TNFα‐induced neutropenia. Among these cases, 8 patients (9%) were on adalimumab, while the majority were using etanercept (73%), or infliximab (18%).^5^ This suggests a potentially lower risk of neutropenia associated with adalimumab compared to other anti‐TNF medications. However, it's important to note that individuals on any form of anti‐TNF therapy are at risk of this uncommon adverse effect. This case report describes a patient with treatment‐resistant Crohn's disease who was initiated on adalimumab and subsequently developed febrile neutropenia.

## 2 CASE REPORT

### 2.1 Case history and examination

A 33‐year‐old male with a confirmed diagnosis of ulcerative colitis (UC) in 2010 who was off treatment presented with a 5‐day history of recurrent lower abdominal pain. The pain was characterized as moderate in severity, dull in nature, and was non‐radiating. This pain was not influenced by food intake but was accompanied by tenesmus (a feeling of incomplete defecation) and the presence of blood mixed with stool. Additionally, the patient experienced a high‐grade fever peaking at 40.5 degrees, along with chills. Notably, there were no accompanying symptoms of nausea or vomiting. The patient denied any recent exposure to sick individuals but did mention recent travel to Oman and Thailand.

Upon physical examination, the patient appeared well without signs of dehydration, jaundice, or pallor. Abdominal examination revealed a soft and lax abdomen that was not distended. Mild tenderness was noted over the lower abdomen, and percussion produced a tympanic sound. No organomegaly was detected.

Initial laboratory assessments demonstrated normal complete blood count (CBC) results, as well as normal kidney function tests (KFT) and liver function tests (LFT). The C‐reactive protein (CRP) level was found to be elevated at 18 mg/L. Rapid malaria antigen testing returned negative results.

Diagnostic procedures, including abdominal imaging and chest X‐rays, did not reveal any significant abnormalities. Subsequent sigmoidoscopy was conducted, which revealed normal digital rectal examination (DRE) findings. However, marked mucosal edema and erythema were observed throughout the rectum, sigmoid colon, and most of the descending colon, along with the absence of a discernible vascular pattern. The mucosal tissue displayed marked erythema, absent vascular pattern, friability, and erosions. This led to the determination of an endoscopic mayo score (EMS) of 2, which signifies moderate disease activity.

The patient was initially managed as a mild UC flare. Treatment was initiated with oral mesalamine at a dosage of 2 g twice daily. Subsequent improvement was noted, and the patient's regimen was transitioned to include both oral and topical 5‐Aminosalicylic Acid (5ASA).

Prior to commencing Adalimumab therapy, the patient underwent pre‐treatment assessments, including a review of HBV serology, a normal chest X‐ray, a negative HIV test, and a non‐reactive QuantiFERON test. The patient's white cell count (WCC) and neutrophil count were both within normal limits.

Adalimumab induction and maintenance therapy were initiated. The patient received an initial dose of 160 mg, followed by subsequent doses of 80 mg after 2 weeks and 40 mg every 2 weeks thereafter, all administered subcutaneously. Significant clinical improvement in the management of UC was observed. However, 1 month later, the patient returned with complaints of fever, generalized weakness, and body pain spanning over 5 days. The patient denied symptoms such as vomiting, altered bowel frequency, constipation, cough, shortness of breath, or dysuria. On examination, the patient was febrile with a documented fever of 38.9 degrees; the remaining vital signs were stable with an unremarkable physical examination.

### 2.2 Investigations

Laboratory investigations revealed a normal hemoglobin level (12.9 gm/dL) and reduced white cell count (1.5 × 10^9^/L) with a diminished neutrophil count (0.2 × 10^9^/L), normal lymphocyte count (2.0 × 10^9^/L), slightly elevated eosinophil count (0.5 × 10^9^/L), and a platelet count of 264 × 10^3^/μL. Also, peripheral smear was done, which only revealed a reduced neutrophil count. Coexistent infection was ruled out, and acute exacerbation of the underlying disease was not observed.

Anti‐nuclear antibody (ANA) testing indicated an elevated titre of 1 in 320, with a homogenous pattern. However, other autoantibodies, including anti‐double‐stranded DNA (anti‐dsDNA), Clithridiate DNA antibody, antineutrophil cytoplasmic antibody (ANCA), and nucleic protein autoantibodies (Ro, La, nRNP, Sm, Jo1, scl70), were not detected excluding any underlying autoimmune condition. No clinical manifestations of lupus were apparent. Lymphocyte subset counts were found to be within normal ranges. Other potential causes of the patient's presentation such as systemic viral infections including HIV, hepatitis B and C serology, CMV, HSV, and EBV were negative. Also, gastrointestinal infections and the use of other medications, including herbal and complementary ones, were ruled out. Nutritional workup, including vitamin B12, serum folate, iron studies, vitamin D, calcium, magnesium, zinc, and copper, was within the normal range.

### 2.3 Outcome and follow‐up

Over time, following the suspension of adalimumab and subsequent monitoring of white cell count, neutropenia exhibited a trend of improvement. A bone marrow examination was not pursued, as neutrophil counts returned to normal after 8 weeks (WCC 8.7 × 10^9^/L, Neutrophils 5.3 × 10^9^/L).

Further administration of Adalimumab was withheld, and the patient's treatment plan was adjusted to include continued use of oral and rectal mesalamine, given the reported stability of the underlying disease.

## 3 CONCLUSION

Anti‐TNF therapies offer substantial progress in managing severe inflammatory conditions, yet potential risks, like rare hematologic complications such as febrile neutropenia, demand attention. Close monitoring of adalimumab‐treated patients for febrile neutropenia is crucial. Understanding causes, predicting risk, and investigating G‐CSF's impact remain vital for optimizing patient safety and outcomes.

## 4 DISCUSSION

Anti‐TNF therapy has become a prominent approach for managing severe and refractory inflammatory bowel disease (IBD) in general and as the first line therapy for moderate to severe Crohn's disease (CD), with applications in both inducing and maintaining disease remission. However, this therapeutic approach is not without challenges. Anti‐TNF antibodies are costly and can yield various side effects. Additionally, a subset of IBD patients, approximately 10%–30%, do not respond adequately to this treatment.^6^ The FDA has approved four anti‐TNF antibodies, namely infliximab, certolizumab, golimumab, and adalimumab, for IBD treatment.^6^ Adalimumab was administered to a patient for IBD treatment, and it was noted that the patient had no history of neutropenia prior to adalimumab usage and did not have any concurrent conditions that could induce neutropenia.

As anti‐TNF therapy heightens the risk of infections, it is recommended to screen individuals scheduled for such therapy for latent tuberculosis and hepatitis B and C infections in order to mitigate the risk of reactivation.^7^ The link between anti‐TNF treatment and cancer development remains contentious, with conflicting trial results: some trials suggest a dose‐dependent rise in malignancy risk with anti‐TNF antibodies,^8^ while others indicate no increase in malignancy risk.^9^, ^10^ Other potential side effects of this therapy encompass injection‐site reactions, allergic responses, lupus‐like symptoms, demyelinating diseases, and elevated liver enzymes and neutrophil counts.^11^ Comprehensive case reports of neutropenia induced by adalimumab are scarce.

Neutrophils, constituting over 70% of white blood cells, play a pivotal role in immune responses against infections and malignancies. Neutropenia, defined as an absolute neutrophil count (ANC) <1.5 × 10^9^/L, is categorized by severity as mild (1.0–1.5 × 10^9^/L), moderate (0.5–1.0 × 10^9^/L), or severe (<0.5 × 10^9^/L).^12^ Neutropenia is commonly linked to viral infections, autoimmune disorders, nutritional deficiencies, and certain congenital conditions like Chediak‐Higashi syndrome, Cohen syndrome, and Griscelli syndrome type 2.^13^ Drug‐induced neutropenia is a known and serious consequence of chemotherapy; however, neutropenia can also result from other medications, including antithyroid drugs (thionamides), anticonvulsants (carbamazepine, phenytoin), clozapine, acyclovir, and various antibiotics.^14^ Notably, anti‐TNF therapy has also been associated with this rare side effect.

The exact mechanism underlying anti‐TNF antibody‐induced neutropenia remains elusive. TNF‐α is integral in a complex cytokine network governing hematopoiesis and regulating granulocyte‐colony stimulating factor (G‐CSF). Consequently, anti‐TNF‐α agents might disrupt bone marrow cytokine balance, potentially leading to bone marrow failure by impeding stem cell differentiation.^5^, ^15^ Neutropenia related to adalimumab has been reported in contexts involving T‐large granular lymphocyte (T‐LGL) expansion,^15^ suggesting the potential involvement of T‐LGL proliferation in the pathogenesis of anti‐TNF‐induced neutropenia through interactions like Fac/Fac or by independently suppressing neutrophil precursors. Another plausible mechanism is the development of antibodies leading to peripheral consumption,^16^ as seen in a case where significant neutropenia and anti‐drug antibody production occurred upon restarting etanercept for the third time without methotrexate in a rheumatoid arthritis patient.^17^

One retrospective cohort study observed that the incidence of severe infections due to neutropenia induced by anti‐TNF‐α therapy was merely 6%.^18^ The notably lower rate of severe and life‐threatening infections in anti‐TNF α‐induced neutropenia compared to other neutropenia causes may indicate a shift of neutrophils from circulation to endothelium adhesion, thereby maintaining availability against infections.^5^ In summary, the pathogenesis involves dysregulation of hematopoiesis microenvironment, LGL expansion, immune‐mediated peripheral consumption, and potentially neutrophil adherence to endothelium.

A key predictor for developing neutropenia under anti‐TNF therapy is a history of neutropenia from another anti‐TNF‐α blocker and a lower baseline neutrophil count, both of which elevate the risk of severe neutropenia.^18^ A retrospective study involving rheumatoid arthritis patients treated with anti‐TNF α demonstrated a correlation between decreased neutrophil counts and improved disease activity.^18^

Managing drug‐induced neutropenia typically entails promptly discontinuing the causative drug if feasible and maintaining a low threshold for treating fever with broad‐spectrum antibiotics. Granulocyte colony‐stimulating factors (G‐CSF) are employed to mitigate infection risk in chemotherapy‐induced neutropenia; however, these agents are not yet sanctioned for other drug‐induced neutropenia. The available data on G‐CSF effects in drug‐induced neutropenia indicate a reduced duration of neutropenia and hospital stay without altering overall mortality.^19^, ^20^ There are no reports on G‐CSF usage in treating anti‐TNF‐induced neutropenia.

## AUTHOR CONTRIBUTIONS

**Ma'In Atallah Mohammad Abumuhfouz:** Conceptualization; data curation; investigation; project administration; supervision; visualization; writing – original draft; writing – review and editing. **Anas Alsadi:** Conceptualization; data curation; writing – original draft; writing – review and editing. **Waseem Fadhl Umer:** Conceptualization; writing – original draft. **Mhd Baraa Habib:** Investigation; visualization.

## FUNDING INFORMATION

This research did not receive any specific grant from funding agencies in the public, commercial, or not‐for‐profit sectors.

## CONFLICT OF INTEREST STATEMENT

The authors report no conflict of interest.

## ETHICS STATEMENT

Ethical Approval was obtained by Medical Research Center (MRC) under ID MRC‐04‐23‐188.

## CONSENT

Written consent was obtained from the patient for publication of this case report and any accompanying images. A copy of the written consent is available for review by the Editor‐in‐Chief of this journal.

## Data Availability

Data can be obtained from the corresponding author upon request.
